# Telehealth nursing interventions for phenotypes of older adults with COPD: an exploratory study

**DOI:** 10.3389/fdgth.2023.1144075

**Published:** 2023-09-21

**Authors:** A. Arnaert, A.M.I. Ahmed, Z. Debe, S. Charbonneau, S. Paul

**Affiliations:** ^1^ Ingram School of Nursing, McGill University, Montreal, QC, Canada; ^2^ Montreal West Island Integrated University Health and Social Service Centre, Montreal, QC, Canada

**Keywords:** telenursing interventions, telemonitoring, older adults, phenotypes, chronic obstructive pulmonary diseases, exploratory research

## Abstract

**Introduction:**

Inconclusive results exist around the effectiveness of telemonitoring for patients with COPD, and studies recommended conducting subgroup analyses to identify patient phenotypes that could benefit from these services. This exploratory study investigated what type of COPD patients were receiving which type of telenursing interventions more frequently using the telemonitoring platform.

**Methods:**

A sample of 36 older adults with COPD were receiving telenursing services for 12 months and were asked to answer five COPD-symptom related questions and submit their vital signs daily.

**Results:**

Findings revealed two phenotypes of older adults for whom the frequency of telenursing calls and related interventions differed. Although no statistically significant differences were observed in participants' GOLD grades and hospitalizations, cluster one participants used their COPD action plan significantly more frequently, and were in frequent contact with the telenurse.

**Discussion:**

It is paramount that further research is needed on the development of patient phenotypes who may benefit from telemonitoring.

## Introduction

Although the use of telehealth technologies and services has accelerated during the COVID-19 pandemic, the provision of remote healthcare is not a new phenomenon (1). Prior to the pandemic, ample evidence exists that telehealth is beneficial for specific uses and patient populations. More specifically, results have shown that the provision of telehealth nursing interventions through a variety of commercial-available telemonitoring platforms for chronic disease management, are associated with a reduction in hospitalizations, emergency room (ER) visits, and mortality (2), improved clinical outcomes, patient perceived health-related quality of life (HRQoL), and overall management of the disease (3).

Nevertheless, for patients with Chronic Obstructive Pulmonary Disease (COPD), an overview of systematic reviews and meta-analyses have demonstrated insufficient evidence to support the effectiveness of telemonitoring interventions on mortality, quality of life (QoL), exercise capacity, exacerbation-related outcomes, and cost-effectiveness (4, 5). A plausible reason for these inconclusive results is, according to the authors, that these synthesized studies on the topic of telemonitoring and COPD vary in scopes, qualities, and outcomes (4). Interestingly, among the suggestions for future research, authors recommended conducting subgroup analyses to identify COPD patient segments or phenotypes that could benefit from telemonitoring (4, 5). More specifically, correctly identifying “who the ideal COPD patient is, at what time patients need telemonitoring, and for how long” is needed as the “one glove fits all” approach is too simplistic for this patient population (6). This recommendation seems evident as COPD is a heterogenous chronic condition, and defining subpopulations from a clinical, physiologic, and radiologic presentation permit to provide personalized healthcare (7–9). Rassouli et al. (10) stated that telehealth is feasible for patients with COPD Global Initiative for Chronic Obstructive Lung Disease (GOLD) score B-D, age ≥40 years of age and have ability to use the technology. A Danish trial study concluded that across the COPD severities, patients with severe COPD GOLD 3 are likely to be the most cost-effective group for telehealth (11). When focusing only on patients with severe airflow limitations and severe COPD exacerbations histories in the preceding year, a systematic review found that adding telemonitoring to usual care reduced unnecessary ER visits but would not prevent hospitalizations due to COPD exacerbations (12). For patients with a past acute COPD history, findings of a meta-analysis indicated that telemonitoring services for a duration of more than 12 months reduces exacerbation-related rehospitalizations and ER visits (13).

Answering the question “what type of COPD patient” may benefit from telehealth is key; however, equally important is defining the “type of telehealth nursing interventions” needed to support COPD self-management. Albeit not for COPD patients, Wakefield et al. (14) specified the type of nursing interventions for patients with diabetes and hypertension using telemonitoring. Most frequently, telenurses are providing lifestyle information and education to patients, and are communicating with the primary care provider. These findings were supported by a systematic review which indicated that telenurses are providing patient education and follow-up care, and are supporting patient empowerment (15). When evaluating a home telehealth nursing service, called “Telesenior”, in Belgium, Arnaert et al. (16, 17) have investigated “what type of homebound older adults needed what type of video-supported telehealth nursing intervention”. Although various segments of seniors could be identified, findings revealed that patients who were older, widowed, lived alone, had financial problems, and used several health and social services benefited significantly from the telehealth nursing interventions. Seniors used the system mainly for social contact, physical and psycho-social health-related questions, financial issues and for social-administrative issues (18). To the authors' knowledge, no studies have defined phenotypes of COPD patients and the associated nursing services offered to these subpopulations. Hence, the purpose of this exploratory study was to investigate, in view of better COPD self-management, what type of COPD patients were receiving which type of telenursing interventions more frequently using the telehealth monitoring platform. The research questions are: (1) With regard to the delivery of appropriate telenursing services, is it still meaningful to speak about “the” COPD patient? and (2) Is it possible to define specific phenotypes of older adults with COPD and draw conclusions about their appropriate telehealth nursing interventions? According to Han et al. (19) a COPD phenotype is defined as “a single or combination of disease attributes that describe the difference between individuals with COPD according to their clinical meaningful outcomes, such as exacerbations, symptoms, etc.”

## Integrated telehealth nursing services

**Figure 1:**
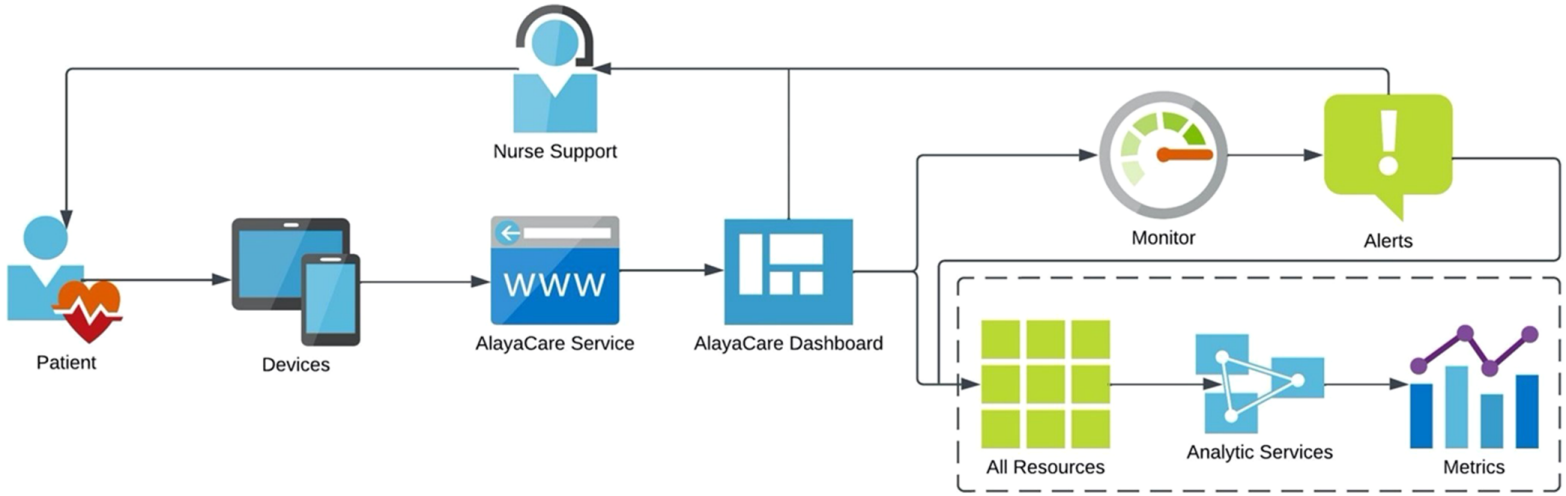
Overview of system flow.

Once patients agreed to participate in the telemonitoring project, the research assistants scheduled a one-hour telephone appointment to install and educate patients on how to use the telemonitoring platform on a mobile device or desktop computer. All patients were provided with a traditional finger pulse oximeter, and those who did not have a mobile device, or their computer was outdated, received a project tablet computer. The AlayaCare telemonitoring platform is a cloud-based application that has the capability of secure, high-quality videoconferencing and remote physiological monitoring, based on input data from patients using peripherals. In this study, COPD patients were asked, according to their individualized care plan, to enter and submit manually to the telenurse, in real time, their clinical data through the platform. More specifically, the protocol required that patients submit both their blood oxygen saturation (SpO_2_) levels and pulse daily during a 12-months period, and to answer five specific COPD-related questions. Participants could enter their pulse and oxygen levels multiple times during the day and night; however, it was required to complete the questions with different answer categories once every day. The following questions were: *How would you describe your level of shortness of breath (SOB) today? How many times did you spit today? What color was your spit today? Was your spit thicker than usual? How often did you cough today?* Tailored patient education material was made available on the system to support them and complement their knowledge regarding lifestyle changes. When a measurement of clinical data was outside of expected patient-specific parameters, the telenurse would contact the patient, provide the necessary interventions, and if needed communicate with the interdisciplinary team at the COPD clinic of a local community hospital. The telenurse and members of the COPD team, two pneumologists and one COPD clinic nurse had access to the telemonitoring platform, however, only the clinic nurse used the system in her clinical practice. After each intervention, all clinicians would complete the interdisciplinary notes on the platform to enhance patient information exchange and continuity of care. The telenurse was a community health nurse who received training on the use of the platform, the COPD disease, and management at the clinic. An overview of the system flow is provided in Figure 1.

## Materials and methods

### Sample and recruitment

This project obtained ethics approval (SMHC # 19-11) in July 2019; however, due to technical platform issues in September 2019, the start of the project was postponed to February 2020. Subsequently, due to the COVID-19 outbreak in March 2020, the patient recruitment process started in June 2020. A sample of 140 patients who had a COPD diagnosis classified as 2 (mild to moderate), 3 (severe) or 4 (very severe) according to the staging system of the GOLD, and were followed by the pneumologists at the local community hospital, were selected by the COPD clinic nurse. Research assistants would phone all patients to explain the project and verify if they are eligible and would be interested to participate. In addition to the clinical eligibility criteria, the patients needed to speak French or English, be willing to be audiotaped and sign the consent. Patients with dementia or suffering from a severe mental illness were excluded. The recruitment of potential participants was done progressively over several months, and a purposive sample of 36 patients were recruited and received telenursing services for a duration of 8 to 12 months.

### Data collection

Patient data were gathered from (a) Patient daily questionnaires, (b) Automated alerts sent to the telemonitoring platform, with high level alerts requiring intervention due to participant input values below threshold for SpO_2_ (less than 88%) and/or heart rate (less than 55 BPM and higher than 110 BPM) values. (c) Telenurses' interdisciplinary notes observing interactions with patients and types of intervention, (d) Participant's self-reported medical history, (e) Participants responses to 6 HRQoL questionnaires at the start of the project (T0 Baseline) and subsequently every 4 months thereafter (T1 Mid Project and T2 End Project). The questionnaires used were (a) the Visual Analog Scale QoL (VAS-QoL) (20), (b) the EQ 5D-5l Questionnaire (21), (c) Hospital Anxiety and Depression Scale (HADS) (22, 23), (d) de Jong-Gierveld Loneliness Scale (24, 25), (e) the Personal Resource Questionnaire (PRQ2000) (26, 27), and (f) St. George's Respiratory Questionnaire (SGRQ) (28, 29). Table 1 presents an overview of responses to each of the questionnaires. At T0 Baseline, all participants completed a socio-demographic survey, and all questionnaires were available in both English and French.

**Table 1:** Overview of participants' responses to the 6 questionnaires.

Questionnaires	Paricipants with at least 1/2/3 complete questionnaires	Total actual^a^/expected^b^ responses	Missing questions^c^
VAS-QOL	36/29/24	89/108	0
EQ 5D-5l	34/29/23	86/108	0
HADS	34/29/25	88/108	1
Loneliness	34/29/24	87/108	11
PRQ2000	33/30/24	87/108	0
SGRQ	33/28/23	84/108	4

^a^ Actual responses from all participants from T0, T1, T2.

^b^ Expected responses if all questionnaires were answered by all participants over T0, T1, T2.

^c^ Missing questions are the total number of questions not answered in a questionnaire.

### Statistical methods and analysis

**Figure 2:**
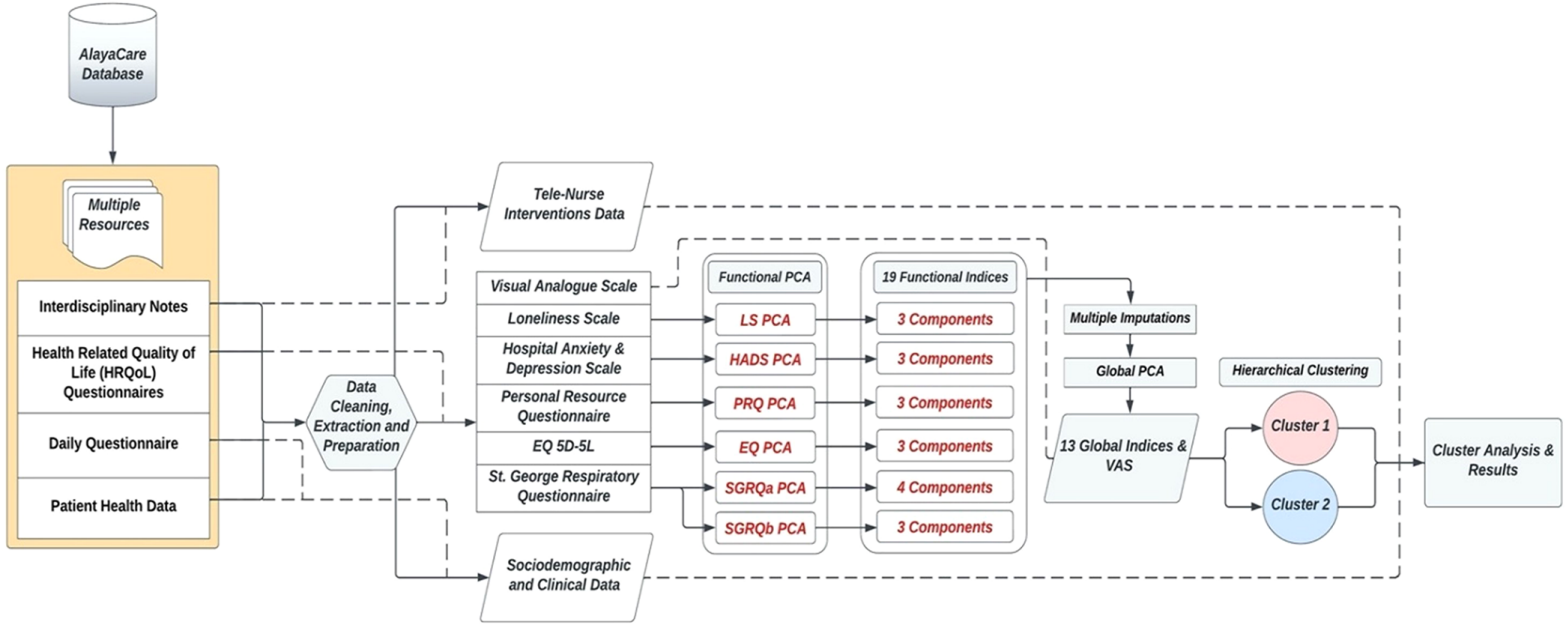
Overview of data flow.

The Principal Components Analysis (PCA) mathematical approach on repeated measures data (T0, T1, T2) was applied to investigate the structure of the properties measured by the assessment scales (30, 31). It allows to reduce the dimensionality of the dataset, while conserving as much as possible the statistical information (32). This paper separates the PCA application into two iterations, as shown in the data flow Figure 2. The summative or Likert scaling model was used to arrive at an aggregate index if the underlying construct was one-dimensional (33). Prior to conducting the PCAs, the answer categories of the scale-items were reversed (ex. a score from 1 to 5) for consistency across instruments, so that high values always implied a more favorable attitude toward some issue. Dealing with missing values prior to the Global PCA was central, and retaining as much of the data as possible, without erroneously affecting the results, was essential given the low study sample size. To overcome this issue without discarding participant records, the regularized PCA mathematical approach was used to replace the missing values (34). The Multiple Imputations (MI) technique (34–36) was applied with the R software packages of FactoMineR (37) and missMDA (38), and used as a sensitivity analysis tool verifying the imputed results. MI allows to derive several other plausible replacements of the missing values, checking for the results' stability. Hierarchical Clustering with Ward's method and the Euclidean distance measure were applied on the standardized values of the global indices to divide the participants into phenotypes (39–41).

Various statistical tests were used to a) compare the phenotypes and b) draw conclusions about the usage of telenursing interventions and its effect on HRQoL. Independent tests were applied to compare phenotypes. The independent *t*-test was used to assess continuous variables, and are presented as mean ± standard deviation with 95% confidence interval. If Levene's test of unequal variances was statistically significant, the Welch's *t*-test two-sided *p*-value was reported instead. The Chi-Square test was applied for categorical variables (presented as a proportion) such as demographic, clinical characteristics and comorbidities. The non-parametric Mann-Whitney *U* test was performed for ordinal global indices, presented as min, median, max, interquartile range; the asymptotic *p*-values were reported. Paired tests were implemented to evaluate the HRQoL between T0, T1, and T2. For all ordinal derived aggregated global indices, the Wilcoxon Signed Rank was used as a non-parametric test, and the paired *t*-test was applied on the continuous VAS scores. For all tests, a *p*-value <0.05 was statistically significant. To further assess the magnitude of differences for both independent and paired tests, the appropriate effect size calculations were implemented based on the type of statistical test applied. Hedge's d was reported for the parametric *t*-tests, while the adjusted calculations for the transformed Cohen's d were reported for the non-parametric Mann-Whitney and Wilcoxon Signed Rank tests (42–44). The statistical package SPSS (45) and online tools of AI-Therapy Statistics (46) and Psychometrica (47) were used for calculations.

## Results

### Functional PCA results

**Figure 3:**
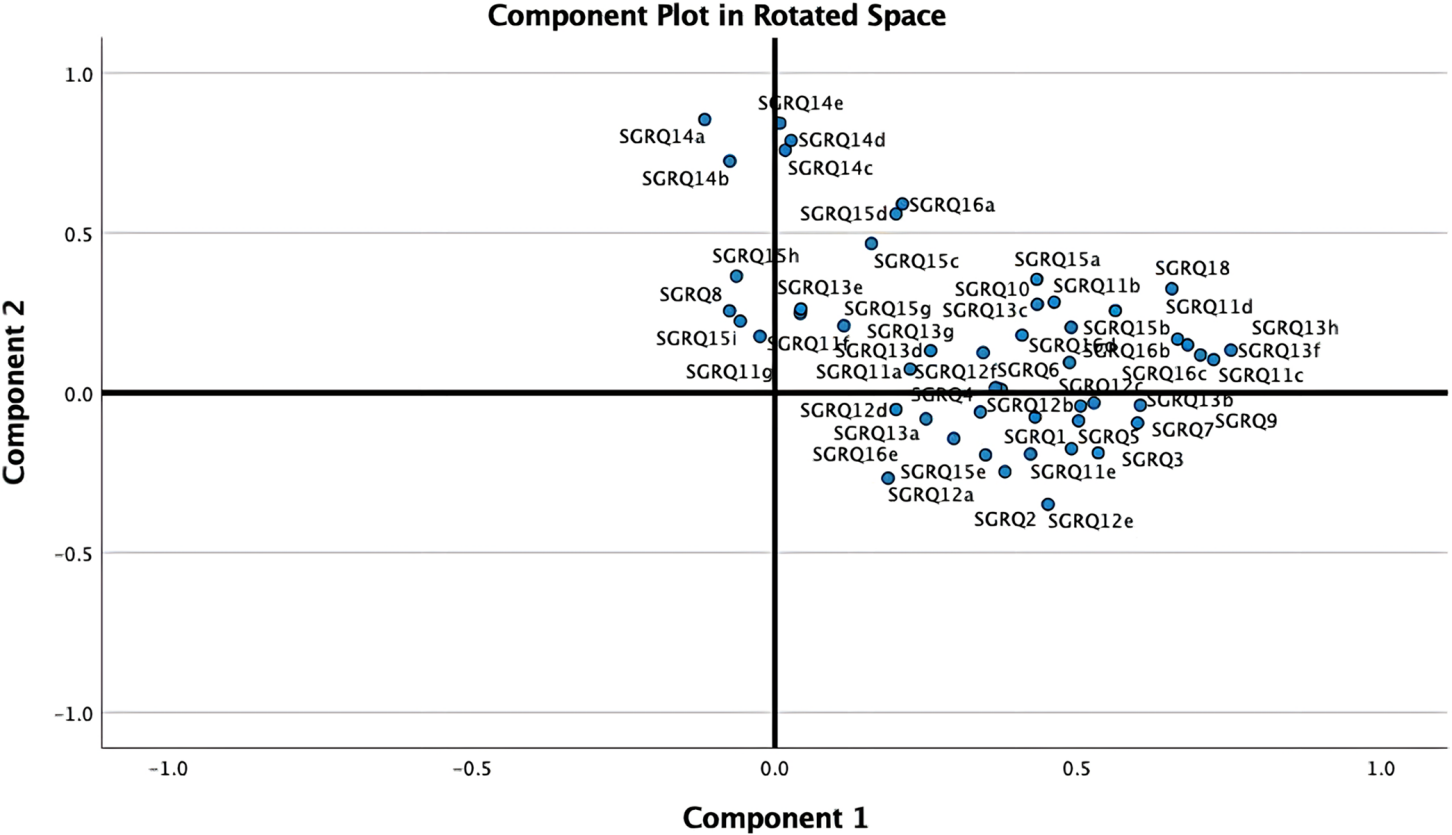
2D projection of the 51-item SGRQ.

As shown in Figure 2, the PCA technique was applied on two iterations. Prior to the second iteration of the Global PCA, the techniques of regularized PCA and MI were used to deal with and justify the replacement of missing values. The initial PCA application was performed on each instrument individually, named “Functional PCA”, which used the patient responses of each of the 5 questionnaires (EQ 5D-5l, HADS, Loneliness, PRQ2000, and SGRQ), consisting of a total of 92 items, and resulted in “19 Functional Indices”. Each questionnaire's Functional PCA application was relatively straightforward, however, the 51-item SGRQ questionnaire, as displayed in a 2D projection in Figure 3, was more challenging, and as such separated into two clinically meaningful parts A and B using an iterative PCA approach and evidence from rotated factor loadings. Part A presents “activities”, and part B the “respiratory symptoms and impact”, as supported by the American Thoracic Society (48).

In order to aggregate all SGRQ activity-related questions (SGRQ Part A) meaningfully, the analyses were based on the findings from Monjazebi et al. (49) and Takechi et al. (50), who identified three sub-categories for Activities of Daily Living (ADL) for patients with COPD and with dementia respectively: Basic-ADL (clothing and bathing), Instrumental-ADL (shopping and food preparation), and Advanced-ADL (hobbies and working). As shown in Table 2, for SGRQ Part A, the 22-items were aggregated into the four functional indices: SGRQ VBADL (Very Basic), SGRQ BADL (Basic), SGRQ IADL (Instrumental), and SGRQ AADL (Advanced).

**Table 2:** Aggregation of the SGRQ part A into 4 functional indices.

Group	Topics	Total # questions	Functional index
1	Mobility, physical ambulation	2	SGRQ-VBADL
2	Bathing, dressing	7	SGRQ-BADL
3	Shopping, entertainment & recreation	4	SGRQ-IADL
4	Housework	2	SGRQ-IADL
5	Sports	2	SGRQ-AADL
6	Moderate to heavy activities (e.g., gardening, shoveling snow, etc.)	4	SGRQ-AADL
7	Job-related activities	1	SGRQ-AADL

**Figure 4:**
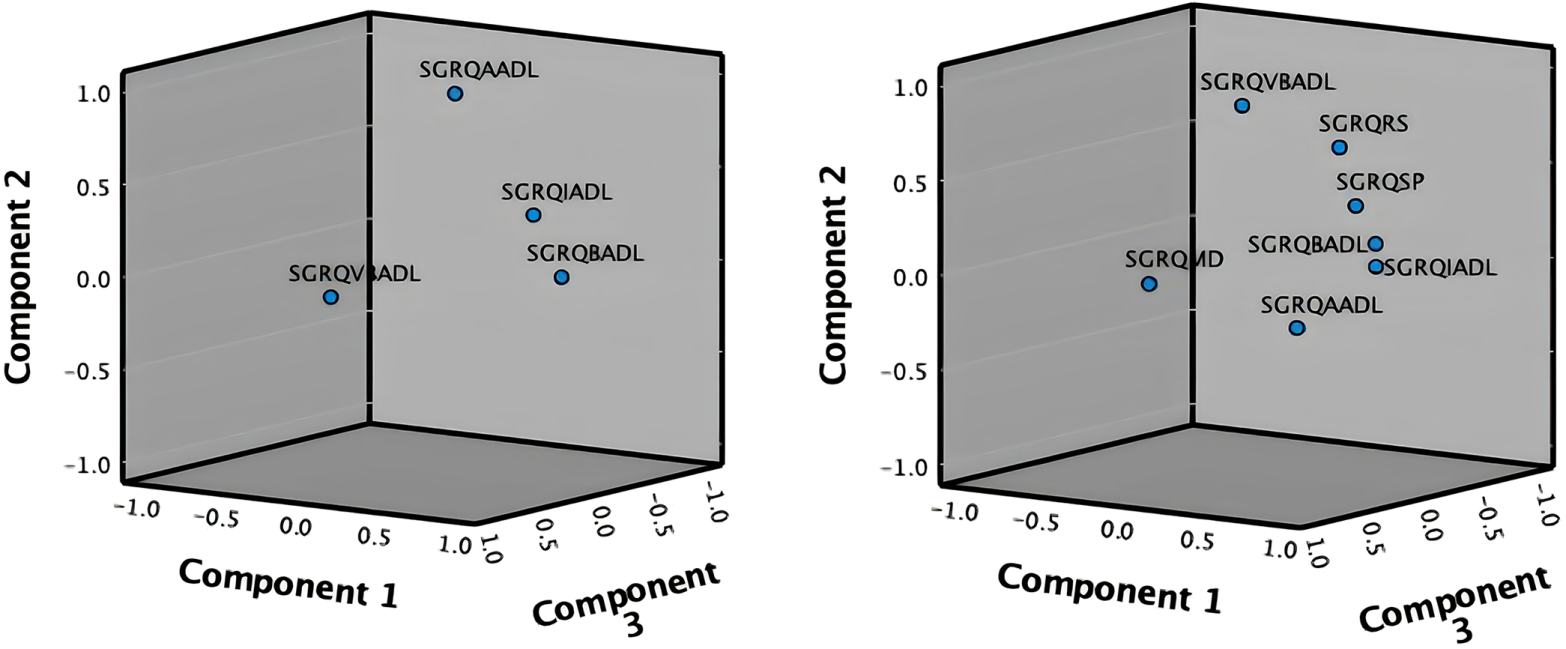
3D projections of SGRQ part A (left), and all 7 SGRQ functional indices (right).

The analysis of Part B of the SGRQ resulted in three distinct functional indices: SGRQ-RS (Respiratory symptoms), SGRQ-SP (COPD Self-perception of Chest Condition), and SGRQ-MD (Medication) (see Table 3). Figure 4 presents all SGRQ functional indices in a 3D visualization.

**Table 3:** Statistical test results of the initial PCA with 19 functional indices.

Questionnaires	KMO value	Bartlett's test	Extracted components / functional indices	Total variance explained
EQ 5D-5l	0.778	<0.001	3	86.240%
HADS	0.826	<0.001	3	59.537%
Loneliness	0.715	<0.001	3	83.286%
PRQ2000	0.847	<0.001	3	63.363%
SGRQ-A	0.747	<0.001	4	79.094%
SGRQ-B	0.720	<0.001	3	87.724%

For all questionnaires, the eigenvalue-one criterion (EV ≥ 1) was used to select the indices; occasionally some indices with an EV < 1 were retained if they provided meaningful insights (50). For example, regarding the indices BADLs and IADLs which had high factor loadings on one component, as reflected in their closeness in Figure 4 left, the decision was made to keep them separately as it was more clinically meaningful, although it resulted in a component with EV < 1. Varimax orthogonal rotation was applied on all questionnaires to aid clarity and interpretability of the relationship between data and the identifiable principal components (51, 52). The resulting Kaiser-Meyer-Olkin (KMO) values were greater than 0.7, and thus classifications are between “middling” to “meritorious” (53). Moreover, as shown in Table 3, Bartlett's test of sphericity was statistically significant with *p*-values <0.001, indicating likely factorizable data.

### Global PCA results

Subsequently to the Functional PCA, a Global PCA was conducted on all “19 Functional Indices” collectively. Instead of looking at each of the questionnaires separately, the goal was now to obtain a global insight of all extracted functional indices combined, leading to “13 new Global Indices” (see Table 4). The KMO value was 0.800 and Bartlett's test *p*-value was <0.001, indicating the applicability of PCA. Varimax orthogonal rotation was used, and the most interpretable and clinically relevant final indices were maintained, and the total variance explained was 93.588%.

**Table 4:** Functional and global indices.

* *	Functional indices	Global indices
	(19 Indices + VAS)	(13 Indices + VAS)
VAS-QOL	General health score (VAS)	VAS
EQ 5D-5l	Anxiety & depression (EQ-AD)	AD *(Anxiety & depression)*
EQ 5D-5l	Physical functioning (EQ-PF)	PF *(Physical functioning)*
EQ 5D-5l	Pain & discomfort (EQ-PD)	PD *(Pain & discomfort)*
HADS	Cheerfulness (HADS-CH)	AD *(Anxiety & depression)*
HADS	Enjoyment (HADS-EN)	AD *(Anxiety & depression)*
HADS	Relaxation & ease (HADS-RE)	RE *(Relaxation & ease)*
Loneliness (LS)	Emotional loneliness (LS-EL)	EL *(Emotional loneliness)*
Loneliness (LS)	Emptiness & rejection (LS-EMRJ)	EL *(Emotional loneliness)*
Loneliness (LS)	Social loneliness (LS-SL)	SL *(Social loneliness)*
PRQ2000	Emotional connection (PRQ-EC)	EL *(Emotional loneliness)*
PRQ2000	Social connection & appreciation (PRQ-SCA)	SL *(Social loneliness)*
PRQ2000	Friendship & belonging (PRQ-FB)	SL *(Social loneliness)*
SGRQ-A	SGRQ-VBADL	VBADL *(Very basic)*
SGRQ-A	SGRQ-BADL	BADL *(Basic)*
SGRQ-A	SGRQ-IADL	IADL *(Instrumental)*
SGRQ-A	SGRQ-AADL	AADL *(Advanced)*
SGRQ-B	Respiratory symptoms (SGRQ-RS)	RS *(Respiratory symptoms)*
SGRQ-B	Self-perception (SGRQ-SP)	SP *(Self-perception)*
SGRQ-B	Medication (SGRQ-MD)	MD *(Medication)*

### Dealing with missing values

**Figure 5:**
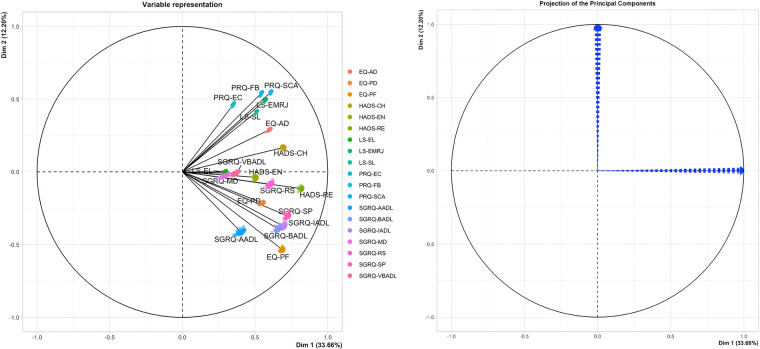
2D plot of MIs on global PCA for 19 functional indices (left) and stability of global PCA (right).

Of the 36 participants, only two answered the VAS (see Table 1), and thus not included in the PCA applications. The remaining 34 patients had answered most questionnaires at baseline (T0); however, as shown in Table 1 the number of participants completing the questionnaires over the three time periods decreased over time, which required the need to recover as much as possible of the data without erroneously influencing the results. Out of the 34 patients, six had responses for some questionnaires but not others, while two of them had multiple missing responses at different timepoints. Thus, there was a total of 8 questionnaires with no responses for all six patients. Table 5 shows an example where Patient 1 has not answered the EQ 5D-5l questionnaire at T0, Patient 2 has not answered the SGRQ at T1, and only answered the EQ 5D-5l at T2. Using the regularized PCA mathematical approach, missing values were therefore replaced for Patient 1 at T0 and Patient 2 at T1. However, following a selection criterion to impute the missing records, Patient 2 scores at T2 were discarded as there was only one complete questionnaire. Otherwise, the imputations for this high number of missing responses were hypothesized to lead to inaccurate results. To conclude, the imputations were applied for 5 patients and a total of 6 questionnaires that fit to the hypothesized MI criterion. To perform a sensitivity analysis and verify the effect of our imputed values on the Global PCA results, the Multiple Imputations (MI) method was used. This method generates several other plausible values for the missing ones, providing information if small or large changes would occur in the Global PCA results. Figure 5 visualizes a 2D projection of the MIs on the Global PCA and shows that if other plausible values were chosen instead of the selected imputed replacements, the Global PCA would still lead to the same results over the first 2 PCA dimensions. This in turn provides confidence in our imputed values and their relatively stable effect on the final 13 Global Indices.

**Table 5:** Multiple imputations criterion.

Multiple imputations criterion			
** **	SGRQ questions	EQ 5D-5L questions	LS/HADS/PRQ questions
Patient 1 (T0)	√	X	√
Patient 1 (T1)	√	√	√
Patient 1 (T2)	√	√	√
Patient 2 (T0)	√	√	√
Patient 2 (T1)	X	√	√
Patient 2 (T2)	X	√	X

### Hierarchical clustering results

**Figure 6:**
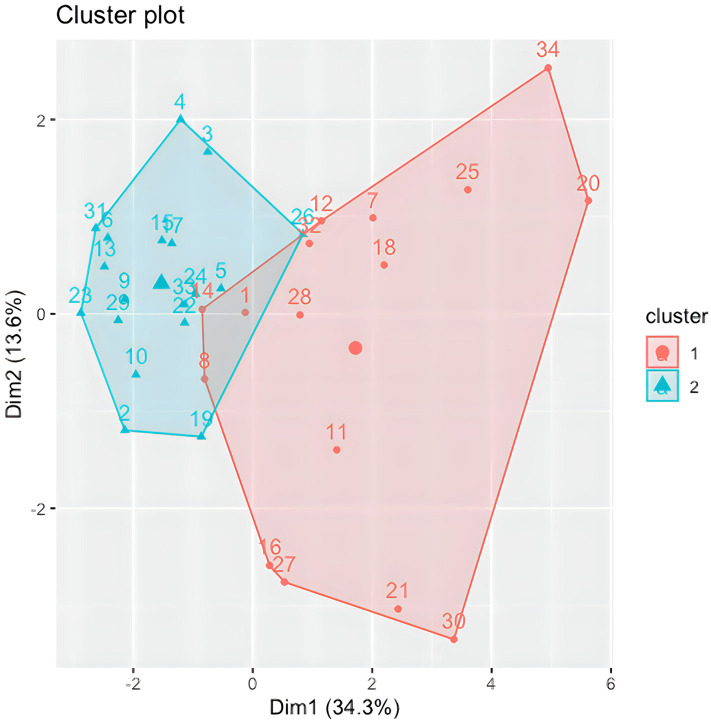
Cluster 2D visualization.

Following the identification of the Global Indices, Agglomerative Hierarchical Clustering was implemented and visualized using the R software (54–57) on the 13 Global Indices and VAS scores for all participants at T0; except for one participant where the T1 scores were used as almost all questionnaires were missing at baseline (and thus not imputed in above MI). The cluster 2D visualization, as shown in Figure 6, represents 2 heterogeneous and balanced clusters with a homogeneous population within each cluster, meaning 2 distinct COPD patients’ phenotypes. Although the elbow plot and silhouette methods have shown that a 3-cluster solution could be justified, a decision was made to retain 2 clusters of participants due to the low number of available data points in this study. Two clusters provide more accurate, robust and generalizable results.

### Statistical tests results

The input data used to compare both phenotypes statistically were participants' socio-demographic and clinical data (comorbidities, GOLD grade, use of action plan and respiratory distress issues), and their T0 Global Indices values. As shown in Table 6, no statistical differences were found between both clusters in terms of participants' socio-demographic characteristics. Although cluster 1 (CL1) (*N* = 16) consisted predominantly of female COPD patients, both clusters comprised mainly of people who were retired, married, lived in the suburbs, have cardiovascular issues, and who quit smoking after years of being heavy smokers. No statistical differences were found between both clusters regarding their COPD GOLD grades; however, statistically significant differences in cough frequencies were observed between both clusters, with CL1 having more frequent coughing and mucus production, and experienced more episodes of shortness of breath compared to cluster 2 (CL2) (*N* = 18). CL1 participants also used their COPD action plan more frequently; however, hospitalizations appeared not to be statistically significant between both clusters.

**Table 6:** Participants socio-demographic and clinical characteristics.

Socio-demographic & clinical characteristics	Cluster 1 (*N* = 16)	Cluster 2 (*N* = 18)	*P*-value	Effect size
Age (mean ± SD; 95% CI)	70.19 ± 5.41; [67.31–73.07]	71.89 ± 9.86; [66.99–76.79]	0.544	–
Gender (*n*, %)				
Female	(13, 81.25%)	(10, 55.56%)	0.110	–
Male	(3, 18.75%)	(8, 44.44%)		
Marital status (*n*, %)				
Divorced	(5, 31.25%)	(2, 11.11%)	0.454	–
Legally married—not separated	(6, 37.50%)	(7, 38.89%)		
Single—never legally married	(2, 12.50%)	(2, 11.11%)		
Widowed	(2, 12.50%)	(5, 27.78%)		
Unknown	(1, 6.25%)	(2, 11.11%)		
Employment status (*n*, %)				
Employed	(2, 12.50%)	(1, 5.56%)	0.303	–
Self-employed	(0, 0.00%)	(1, 5.56%)		
Retired	(12, 75.00%)	(15, 83.32%)		
Unable to work	(2, 12.50%)	(0, 0.00%)		
Unknown	(0, 0.00%)	(1, 5.56%)		
Living environment (*n*, %)				
In a suburb	(15, 93.75%)	(17, 94.40%)	0.365	–
In a city center	(1, 6.25%)	(0, 0.00%)		
In rural area	(0, 0.00%)	(1, 5.60%)		
Smoking behavior (*n*, %)				
Heavy smoker (at least 15 cigarettes a day)	(4, 25.00%)	(2, 11.11%)	0.443	–
Light smoker (less than 10 cigarettes a day)	(1, 6.25%)	(0, 0.00%)		
Non-daily smoker	(0, 0.00%)	(1, 5.56%)		
Used to smoke, but have quit	(10, 62.50%)	(11, 61.11%)		
Unknown	(1, 6.25%)	(4, 22.22%)		
Cardiovascular disease (*n*, %)	(10, 62.50%)	(12, 66.67%)	0.800	–
Endocrine disease (*n*, %)	(2, 12.50%)	(5, 27.78%)	0.271	–
Rheumatic & musculoskeletal (*n*, %)	(4, 25.00%)	(1, 5.56%)	0.110	–
Malignancies (*n*, %)	(2, 12.50%)	(2, 11.11%)	0.900	–
Gastrointestinal disease (*n*, %)	(3, 18.75%)	(0, 0%)	0.054	–
Other respiratory diseases (*n*, %)	(2, 12.50%)	(1, 5.56%)	0.476	–
GOLD Grade (*n*, %)				
GOLD 2	(4, 25.00%)	(3, 16.67%)	0.615	–
GOLD 3	(8, 50.00%)	(12, 66.66%)		
GOLD 4	(4, 25.00%)	(3, 16.67%)		
Daily questionnaire respiratory symptoms [min, max] (Mean score per patient) (mean ± SD; 95% CI)	Cluster 1 (*N* = 13)	Cluster 2 (*N* = 15)	** **	** **
Cough [1, 4]	3.00 ± 0.18; [2.89–3.11]	3.34 ± 0.36; [3.14–3.54]	**0**.**005**	**1.099**
Shortness of breath [1, 5]	2.84 ± 1.16; [2.14–3.55]	3.85 ± 0.92; [3.34–4.36]	**0**.**017**	**0.941**
Spit color [1, 5]	3.95 ± 0.49; [3.65–4.25]	4.00 ± 0.37; [3.80–4.21]	0.754	0.116
Spit frequency [1, 5]	3.79 ± 0.77; [3.32–4.25]	3.82 ± 0.76; [3.40–4.24]	0.904	0.045
Spit thickness [1, 2]	1.95 ± 0.06; [1.91–1.98]	1.92 ± 0.18; [1.83–2.02]	0.648	0.170

The bold values indicated the significant values.

In terms of participants' HRQoL (see Table 7), CL1 participants had significantly lower scores in their physical functioning and ability to perform basic, instrumental and advanced-ADL. They experienced more pain and discomfort, had significantly more signs of respiratory distress, had a low self-perception, and found it difficult to relax and be at ease because of their severe chronic condition. No statistical differences in mental global indices were evident at T0 or any other study time periods, although for the indices “Anxiety & Depression”, and for “Social Loneliness”, CL1 scores were lower. To further note, statistically significant differences between the two clusters were observed in some of the 13 Global Indices in each of the study periods (T0, T1, T2), as presented in Table 7. Moreover, when comparing the Global Indices and VAS scores across the time periods in terms of health improvements, minor statistically significant differences were observed (see Table 8). Despite receiving telenursing services, statistical significance with some health deterioration over time was observed for CL1 for the indices “Physical Functioning” and “Relaxation & Ease” between T1 and T2, and regarding the “Advanced-ADL” indices for CL2 between T0 and T2. Otherwise, the other 11 indices for CL1 and 12 indices for CL2 show no statistical significances between study time periods, which may also suggest the relative stability over time in HRQoL for each cluster.

**Table 7:** Global indices and VAS scores for T0, T1, and T2.

** **	T0 (Baseline)				T1 (Mid-follow-up)				T2 (Final follow-up)			
Global index [min, max] (mean ± SD; 95% CI) or [min, median, max, IQR]	Cluster 1	Cluster 2	** **	** **	Cluster 1	Cluster 2	** **	** **	Cluster 1	Cluster 2	** **	** **
	(*N* = 15^a^)	(*N* = 18)			(*N* = 13^b^)	(*N* = 15)			(*N* = 13)	(*N* = 13)		
General health score (VAS) [0, 100]	69.63 ± 15.84; [61.18–78.07]	74.39 ± 16.97; [65.95–82.83]	0.406	0.283	62.08 ± 20.05; [49.34–74.82]	73.00 ± 9.22; [67.89–78.11]	0.102	0.706	63.85 ± 18.61; [52.60–75.09]	71.92 ± 8.05; [67.06–76.79]	0.170	0.545
Very basic ADLs [2, 4]	[3, 4, 4, 1]	[4, 4, 4, 0]	**0.021**	**0.465**	[3, 4, 4, 0]	[4, 4, 4, 0]	0.122	0.263	[3, 4, 4, 1]	[3, 4, 4, 0]	0.286	0.264
Basic ADLs [7, 14]	[7, 8, 11, 3]	[8, 11, 14, 2]	**<0.001**	**1.432**	[7, 7, 11, 2]	[8, 10, 14, 3]	**0.002**	**1.420**	[7, 7, 10, 2]	[8, 11, 13, 3]	**<0.001**	**2.195**
Instrumental ADLs [6, 12]	[6, 8, 9, 3]	[7, 9, 10, 0]	**<0.001**	**1.386**	[6, 8, 9, 3]	[7, 9, 11, 1]	**0.031**	**0.807**	[6, 7, 9, 3]	[7, 9, 10, 0]	**0.005**	**1.148**
Advanced ADLs [7, 15]	[7, 9, 10, 2]	[7, 9, 12, 1]	**0.021**	**0.808**	[7, 8, 10, 2]	[7, 9, 11, 1]	0.077	0.670	[7, 8, 13, 2]	[7, 9, 10, 2]	0.071	0.728
Physical functioning [3, 15]	[5, 11, 13, 4]	[10, 13, 15, 3]	**0.002**	**1.225**	[6, 10, 14, 5]	[10, 14, 15, 4]	**0.007**	**1.170**	[6, 9, 13, 5]	[10, 14, 15, 2]	**<0.001**	**1.928**
Pain & discomfort [1, 5]	[2, 3, 5, 1]	[3, 4, 5, 1]	**0.013**	**0.897**	[2, 4, 5, 1]	[2, 5, 5, 1]	**0.012**	**0.991**	[2, 3, 5, 1]	[3, 5, 5, 1]	**0.019**	**0.973**
Respiratory symptoms [14, 49]	[21, 27, 33, 7]	[19, 35, 42, 5]	**<0.001**	**1.542**	[21, 27, 36, 5]	[25, 32, 41, 6]	**0.001**	**1.503**	[18, 26, 37, 10]	[24, 32, 41, 7]	**0.009**	**1.195**
Self-perception [10, 24]	[12, 15, 17, 3]	[13, 19, 22, 2]	**<0.001**	**2.305**	[12, 14, 20, 6]	[17, 20, 22, 3]	**<0.001**	**1.591**	[11, 14, 20, 3]	[15, 19, 21, 4]	**<0.001**	**1.875**
Medication [5, 14]	[7, 9, 14, 1]	[8, 9, 14, 0]	0.351	0.293	[6, 9, 14, 1]	[8, 9, 14, 6]	0.168	0.512	[6, 9, 14, 1]	[8, 9, 14, 3]	0.319	0.368
Relaxation & ease [5, 20]	[8, 13, 18, 2]	[13, 16, 19, 3]	**0.002**	**1.225**	[8, 14, 19, 5]	[12, 16, 20, 3]	**0.016**	**1.015**	[7, 12, 16, 4]	[13, 15, 19, 3]	**<0.001**	**1.849**
Anxiety & depression [10, 41]	[23, 35, 41, 10]	[29, 37, 41, 9]	0.216	0.438	[22, 32, 40, 10]	[27, 37, 41, 7]	0.127	0.600	[23, 34, 40, 12]	[29, 36, 40, 6]	0.129	0.621
Emotional loneliness [8, 44]	[25, 37, 44, 11]	[19, 39.5, 42, 10]	0.253	0.405	[24, 37, 42, 9]	[18, 37, 42, 7]	0.487	0.263	[14, 32, 44, 14]	[16, 37, 43, 8]	0.316	0.400
Social loneliness [13, 79]	[28, 60, 79, 20]	[43, 60.5, 76, 10]	0.664	0.151	[32, 60, 76, 21]	[44, 59, 75, 13]	0.872	0.061	[19, 60, 78, 32]	[44, 58, 76, 13]	0.980	0.010

^a^ VAS had *N* = 16 responses at T0.

^b^ VAS had *N* = 12 responses at T1.

The bold values indicated the significant values.

**Table 8:** Global indices and VAS scores across time periods.

** **	Cluster 1			Cluster 2		
Global index	*p*-value (effect size) for T0 vs. T1	*p*-value (effect size) for T1 vs. T2	*p*-value (effect size) for T0 vs. T2	*p*-value (effect size) for T0 vs. T1	*p*-value (effect size) for T1 vs. T2	*p*-value (effect size) for T0 vs. T2
General health score	0.147 (0.435)	0.889 (0.040)	0.161 (0.401)	0.131 (0.402)	0.909 (0.033)	0.180 (0.382)
Very basic ADLs	0.180 (0.570)	0.564 (0.228)	0.705 (0.155)	»1.00 (0.000)	0.317 (0.417)	0.317 (0.400)
Basic ADLs	0.705 (0.155)	0.059 (0.798)	0.066 (0.811)	0.863 (0.063)	0.861 (0.071)	0.618 (0.197)
Instrumental ADLs	0.257 (0.476)	0.458 (0.295)	0.655 (0.183)	0.480 (0.260)	»1.00 (0.000)	0.317 (0.400)
Advanced ADLs	0.564 (0.237)	0.783 (0.108)	0.518 (0.266)	0.221 (0.459)	0.589 (0.222)	**0.020** (**1.029)**
Physical functioning	0.952 (0.024)	**0.020** (**1.029)**	0.366 (0.376)	0.534 (0.229)	0.366 (0.376)	0.856 (0.071)
Pain & discomfort	0.705 (0.155)	0.414 (0.324)	0.739 (0.136)	0.380 (0.325)	0.739 (0.136)	0.589 (0.213)
Respiratory symptoms	0.811 (0.098)	0.231 (0.483)	0.358 (0.382)	0.608 (0.188)	0.592 (0.220)	0.624 (0.193)
Self-perception	0.260 (0.473)	0.297 (0.418)	0.964 (0.018)	0.522 (0.235)	0.084 (0.752)	0.465 (0.290)
Medication	0.380 (0.364)	0.914 (0.042)	0.854 (0.075)	0.172 (0.514)	0.655 (0.183)	0.730 (0.136)
Relaxation & ease	0.192 (0.552)	**0.018** (**1.053)**	0.301 (0.431)	0.196 (0.486)	0.877 (0.063)	0.684 (0.160)
Anxiety & depression	0.655 (0.183)	0.964 (0.018)	0.823 (0.092)	0.620 (0.182)	0.256 (0.477)	0.724 (0.139)
Emotional loneliness	0.559 (0.240)	0.129 (0.623)	0.592 (0.220)	0.844 (0.072)	»1.00 (0.000)	0.532 (0.247)
Social loneliness	0.533 (0.257)	0.366 (0.360)	0.969 (0.016)	0.733 (0.125)	0.448 (0.313)	0.594 (0.210)

The bold values indicated the significant values.

All participants called the telehealth nurse for various reasons; however, for analysis purposes the nursing interventions provided were categorized in “General, Teaching, Evaluation and Follow-up”. Table 9 provides a list of interventions under each category. Important to notice that during each call, the telenurse may have provided one or multiple interventions. For instance, if an evaluation is the topic of discussion, either anxiety, respiratory and/or pain assessments could have been conducted in a single call. Compared to CL2, participants in CL1 have used the telehealth system significantly more frequently across all categories and are represented in the derived variable “Intensity of Care” (*p* = .011). As shown in Table 9, they have significantly used the system more for advice (*p* = .003), teaching breathing exercises (*p* = .002) and how to cough effectively (*p* = .028), assessment of respiratory symptoms (*p* = .003), verification of daily clinical responses (*p* = .002), vital signs (*p* = .001), correct usage of breathing technique (*p* = .014), and follow-ups after a high alert was signaled through the telemonitoring system (*p* = .001). The high number of post-alert follow-ups by the telenurse indicates that CL1 participants' SpO_2_ and/or heart rate values were more frequently outside their personalized parameters for those vital signs, which is captured in the variable “Number of High-Level Alerts”, and statistically significant higher for CL1 (*p* = .015). Important to notice is that the high-level alerts for participant 21 were classified as a “very extreme outlier”, and were removed from the analysis in order not to skew the results; yet, CL1 still had a higher statistically significant difference in the number of high-level alerts.

**Table 9:** Telenursing interventions.

Telenursing interventions	Cluster 1 (*N* = 16)	Cluster 2 (*N* = 18)	** **	** **
Number of responses to daily questionnaires	214.13 ± 104.94; [158.21–270.04]	158.17 ± 96.87; [110.00–206.37]	0.116	0.542
Number of telenursing calls	17.88 ± 8.33; [13.43–22.32]	8.61 ± 6.44; [5.41–11.81]	**<0** **.** **001**	**1** **.** **224**
General	4.75 ± 3.92; [2.66–6.84]	1.72 ± 1.60 [0.93–2.52]	**0** **.** **009**	**1** **.** **009**
Advisory call	2.06 ± 1.98; [1.01–3.12]	0.28 ± 0.58; [0.00–0.56]	**0** **.** **003**	**1** **.** **227**
General COPD info	1.38 ± 2.39; [0.10- 2.65]	0.50 ± 0.92; [0.04–0.96]	0.160	0.483
Weekly checkup	0.31 ± 0.48; [0.06–0.57]	0.17 ± 0.38 [0.00–0.36]	0.332	0.331
Notification of action plan change	0.44 ± 0.73 [0.05–0.83]	0.11 ± 0.32; [0.00–0.27]	0.113	0.578
Other	1.63 ± 1.50; [0.83–2.42]	1.11 ± 1.32; [0.45–1.77]	0.296	0.356
Teaching	6.81 ± 4.65; [4.33–9.29]	3.50 ± 2.96; [2.03–4.97]	**0** **.** **017**	**0** **.** **841**
Treatment / action plan	3.25 ± 3.36; [1.46–5.04]	1.61 ± 1.98; [0.63–2.59]	0.088	0.590
COPD self-management	1.88 ± 1.86; [0.89–2.86]	0.89 ± 1.37; [0.21–1.57]	0.085	0.596
Breathing exercises	1.94 ± 1.12; [1.34–2.54]	0.72 ± 0.96; [0.25–1.20]	**0** **.** **002**	**1** **.** **142**
Platform usage	1.50 ± 0.97; [0.99–2.01]	1.11 ± 0.58; [0.82–1.40]	0.174	0.483
Physical activity	1.19 ± 1.47; [0.40–1.97]	0.67 ± 0.91; [0.22–1.12]	0.217	0.422
Cough effectively	1.06 ± 1.00; [0.53–1.59]	0.39 ± 0.70; [0.04–0.74]	**0** **.** **028**	**0** **.** **772**
Anxiety management	0.81 ± 1.28; [0.13–1.49]	0.28 ± 0.70; [0.00–0.61]	0.130	0.522
Nutrition	0.31 ± 0.48; [0.06–0.57]	0.11 ± 0.32; [0.00–0.27]	0.168	0.487
Evaluation	12.56 ± 6.53 [9.08–16.04]	6.28 ± 5.28 [3.65–8.90]	**0** **.** **004**	**1** **.** **040**
Respiratory assessment	12.44 ± 6.37; [9.05–15.83]	6.17 ± 5.01; [3.68–8.66]	**0** **.** **003**	**1** **.** **077**
Anxiety assessment	1.13 ± 1.75; [0.19–2.06]	0.56 ± 1.15; [0.00–1.13]	0.265	0.381
Pain assessment	0.75 ± 1.48; [0.00–1.54]	0.17 ± 0.38; [0.00–0.36]	0.145	0.541
Follow-up	15.50 ± 7.18 [11.67–19.33]	7.33 ± 6.15 [4.28–10.39]	**0** **.** **001**	**1** **.** **199**
Verification of daily clinical responses	12.25 ± 7.11; [8.46–16.04]	5.06 ± 5.04; [2.55–7.56]	**0** **.** **002**	**1** **.** **152**
Verification of vital signs	12.69 ± 7.11; [8.90–16.47]	5.22 ± 5.20; [2.64–7.81]	**0** **.** **001**	**1** **.** **182**
Post-alert follow-up	5.00 ± 3.78; [2.99–7.01]	1.06 ± 2.01; [0.05–2.06]	**0** **.** **001**	**1** **.** **295**
Call if no data received	2.75 ± 2.57; [1.38–4.12]	2.22 ± 2.63; [0.92–3.53]	0.559	0.198
Emergency contact if no response	0.38 ± 0.72; [0.00–0.76]	0.00 ± 0.00; [0.00–0.00]	0.054	0.744
Verification of breathing technique	1.00 ± 0.89; [0.52–1.48]	0.33 ± 0.59; [0.04–0.63]	**0** **.** **014**	**0** **.** **868**
Data fill-in	0.81 ± 0.40; [0.60–1.03]	0.44 ± 0.51; [0.19–0.70]	**0** **.** **026**	**0** **.** **775**
Intensity of care [derived ordinal scale 1 to 7]	[1, 3.5, 7, 2]	[1, 2, 5, 2]	**0**.**011**	**0**.**906**
Average duration of call	17.50 ± 3.46; [15.65–19.35]	18.06 ± 4.19; [15.97–20.14]	0.679	0.140
Number of high-level alerts *(outliner removed*^a^*)*	4.93 ± 5.34; [1.84–8.01]	0.88 ± 1.63; [0.008–1.74]	**0**.**015**	**1**.**030**
Average yearly action plan use reported by participants^b^	4.13 ± 4.34; [1.73–6.54]	1.56 ± 1.75; [0.63–2.50]	**0**.**046**	**0**.**766**
Average yearly hospitalizations reported by participants^c^	4.53 ± 6.01; [1.20–7.86]	3.94 ± 4.63; [1.47–6.40]	0.758	0.109

^a^ *N* = 14 Patients in Cluster 1 (after removing outlier) and *N* = 16 Patients in Cluster 2; removing patients with less than 10 responses to the daily questionnaire throughout the whole study period as to not skew the results.

^b^ *N* = 15 Patients in Cluster 1 and *N* = 16 Patients in Cluster 2 reported their average use.

^c^ *N* = 15 Patients in Cluster 1 and *N* = 16 Patients in Cluster 2 reported their average use.

The bold values indicated the significant values.

Finally, statistically significant results were mostly complemented with large effect sizes, classified by values equal to or larger than 0.8 (58, 59). Thus, the differences between the two clusters in terms of clinical dissimilarities as well as the disparities in the utilization of the telehealth system is mostly of high magnitude (effect size ≥0.8), as well, results appear to not be due to random chance on the 95% confidence level (*p*-value <0.05). This gives confidence in our results' clinical meaningfulness (60–63) and warrants further investigation of the derived phenotypes and their telenursing usage.

## Discussion

Results of this first-time exploratory study revealed 2 phenotypes of older adults with COPD for whom the frequency of telenursing calls and related interventions differed. Although no statistically significant differences were observed in participants' GOLD grades and number of hospitalizations, CL1 used their COPD action plan significantly more frequently when experiencing an exacerbation. Evidently, due to the high-level of telemonitoring alerts, these CL1 patients were in frequent contact with the telenurse who provided a range of interventions, including education, guided self-management, and pulmonary rehabilitations. Though more research is needed, one may assume that older adults with a similar profile to those in CL1 are “good” candidates for telehealth nursing services. Despite these intriguing results, some points warrant further discussion: (1) Patient risk profiles required for the provision of sustainable telemonitoring services; and (2) Clinical meaningful phenotypes with small study samples.

### Patient risk profiles required for the provision of sustainable telemonitoring services

Although the potential widespread usage of telemonitoring for patients with chronic conditions was part of a long-term plan for many healthcare systems, the COVID-19 pandemic has forced the acceleration of this process (64), and has supported the break-down of many historical barriers such as patients and clinicians’ beliefs about virtual care, lack of reimbursement, cybersecurity, and digital interoperability (65). New analyses have shown that the uptake of virtual care at the early start of COVID-19 in April 2020 was 78 times higher compared to February 2020; yet utilization levels have largely stabilized (66). This implores the question, if telemonitoring has shown a potential improve of patient outcomes, and a reduction in hospital readmissions during COVID-19, why are these services not widely implemented? Undoubtedly, the sustainable uptake of new technology-related services, such as telemonitoring, and its widespread diffusion require integration in the healthcare systems (67) and alignment with existing clinical practices (68). Existing hurdles for wider application post-COVID are cost, lack of evidence-based clinical guidelines and protocols, and according to Danne et al. (64) the absence of “telehealth patient risk profiles”. Interestingly, the authors (64) emphasized that “the future of virtual healthcare is not simply about keeping people away from hospitals, yet it is equally about knowing who should be asked to come to the clinic and when”. In the same vein, Choudhary et al. (69) proposed a multi-factorial risk stratification and risk-based follow-up system to help prioritize patients post-COVID for virtual or face-to-face appointments. An identical telehealth management and risk-stratification approach of older adults with chronic heart failure was implemented successfully by Orso et al. (70) during COVID-19, and they recommended the calculation of patient risk groups in the post-pandemic world to appropriately manage the care of patients remotely and dedicate the necessary resources. Results of this study support these recommendations and strongly encourage researchers to further develop risk algorithms using advanced analytics to define phenotypes of COPD telemonitoring patients to foster the provision of tailored nursing services to subsets of patients.

In recent years, the growing use of machine learning algorithms and cluster analysis techniques have been instrumental to identify COPD phenotypes in general. The systematic review conducted by Nikolaou et al. (71) found a substantial heterogeneity in both the numbers and the clinical features presented in the many COPD phenotypes. The COPD phenotype of “older patients with frequent exacerbations, a high rate of comorbidities (such as cardiovascular diseases) and a fast decline in lung function and QoL” is closely aligned with the characteristics of CL1 participants. Although patients with acute exacerbations often reported a poor HRQoL (72), minor statistically significant changes in participant's QoL were observed in both our study clusters. In terms of the clinical features used to define the COPD phenotypes, no standardization exists; however, Nikolaou et al. (71) suggested to complement risk-stratification models based on clinical severity, as was performed in this study to define both clusters of COPD patients, with other determinants, such as physiological characteristics (e.g., age, body mass index, waist circumference), comorbidities, pulmonary function tests, biomarkers, and genetic variants. In addition, Parikh et al. (73) advised including social, economic, behavioral, and environmental determinants of health when defining phenotypes. Overall, further research is needed to develop COPD phenotypes in general, and more specifically on the development of COPD phenotypes who may benefit from telemonitoring to support the move towards “precision health” where “the right telenursing interventions are provided to the right patients, at the right time in their disease trajectory”.

### Clinical meaningful phenotypes with small study samples

Regardless of the clustering method used, Nikolaou et al. (71) recommended the use of prospective longitudinal data with large samples to develop clinically meaningful COPD-derived phenotypes as clustering methods are data driven techniques. Although there is a known bias of estimates with small samples, there is no theorem supporting the rule of thumb for the size of the dataset for cluster analysis; yet hierarchical clustering works well for small datasets (74). When dealing with small study samples, the key is to carefully interpret the results (75). Despite the many data decisions and operations conducted, the meticulous methodological approach used in this study despite the small sample size has permitted to discover 2 phenotypes of older adults with COPD, with CL1 using the telemonitoring system more evidently. While unconventional for many, the methodology used, separate PCA iterations, carefully imputing missing values, and utilizing the correct clustering technique, has allowed for accuracy, clinical relevance, interpretability, and reproducibility of methods and results. In fact, utilizing both mathematical and statistical results, as well as field expertise of the researchers and clinical literature, has allowed this study to assess and produce clinically meaningful phenotypes using a small dataset. Although the aim for this study was to recruit a sample of 140 patients for high statistical power, unforeseen circumstances, such as the emergence of the COVID-19 pandemic, has led to a drastic reduction in the enrolled participants. The importance, however, of following a structured methodology and reporting not just the *p*-values but also the effect sizes is paramount (61, 62). Given the statistical significance and high effect sizes in our results (76), and although this should not be taken for population effect sizes; it is still large enough to provide evidence and warrant further investigation of the resulting COPD phenotypes.

## Limitations

Several limitations must be addressed. First, MI has emerged as a popular technique for dealing with missing data issues (77); yet there is no substitute for actual participant responses. Second, although statistically significant results were coupled with high effect sizes, large standard deviations still existed in the data due to the small sample size. Third, some technical platforms challenges may have caused that some telemonitoring alerts were not processed correctly, for example the very extreme outlier. Fourth, 3 different telenurses were employed during this study, and a variation in their registration of nursing interventions was observed. In addition, over time fewer nursing interventions were being input into the system, which may have caused some discrepancies in the cluster comparisons. Lastly, in terms of generalization of the results, the clinical interpretations and decisions made to aggregate the various questionnaires into meaningful indices may differ if this study would be replicated by other clinicians and researchers.

## Conclusion

Using a meticulous methodological approach, supported by statistically significant results and large effect sizes, the results showed the identification of 2 COPD phenotypes, with participants in CL1 needing the telenursing services more evidently compared to those in CL2. Concluding, as regards the delivery of appropriate, cost-effective, and sustainable telenursing services, it is paramount that further research is needed to develop COPD patients' phenotypes who may benefit from telemonitoring to support the provision of customized care.

## Data Availability

The datasets generated and/or analyzed during the current study are available from the corresponding author on reasonable request.

## References

[1] Claypool B. Telemedicine and COVID-19: 6 tips to ace your first visit. Mental Health Weekly. (2020) 30(17):5–6. 10.1002/mhw.32337

[2] Paré G Poba-Nzaou P Sicotte C. Home telemonitoring for chronic disease management: an economic assessment. Int J Technol Assess Health Care. (2013) 29(2):155–61. 10.1017/S0266462313000111 23514722

[3] Paré G Jaana M Sicotte C. Systematic review of home telemonitoring for chronic diseases: the evidence base. J Am Med Inform Assoc. (2007) 14(3):269–77. 10.1197/jamia.M2270 17329725 PMC2244878

[4] Li X Xie Y Zhao H Zhang H Yu X Li J. Telemonitoring interventions in COPD patients: overview of systematic reviews. BioMed Res Int. (2020) 2020:5040521. 10.1155/2020/5040521 32016115 PMC6988702

[5] Barbosa MT Sousa CS Morais-Almeida M Simões MJ Mendes P. Telemedicine in COPD: an overview by topics. COPD. (2020) 17(5):601–17. 10.1080/15412555.2020.1815182 32892650

[6] Vitacca M Montini A Comini L. How will telemedicine change clinical practice in chronic obstructive pulmonary disease? Ther Adv Respir Dis. (2018) 12:1–19. 10.1177/1753465818754778 PMC5937158 29411700

[7] Rennard SI. COPD Heterogeneity: what this will mean in practice. Respir Care. (2011) 56(8):1181–7. 10.4187/respcare.01419 21801580

[8] Barrecheguren M Miravitlles M. COPD heterogeneity: implications for management. Multidiscip Respir Med. (2016) 1(1). 10.1186/s40248-016-0053-4 PMC4794904 26989488

[9] Park TS Lee JS Seo JB Hong Y Lee SW Oh YM Phenotyping of chronic obstructive pulmonary disease: heterogeneity and its clinical relevance. Curr Respir Care Rep. (2012) 1(3):189–98. 10.1007/s13665-012-0021-1

[10] Rassouli F Pfister M Widmer S Baty F Burger B Brutsche MH. Telehealthcare for chronic obstructive pulmonary disease in Switzerland is feasible and appreciated by patients. Respiration. (2016) 92(2):107–13. 10.1159/000448377 27553807

[11] Udsen FW Lilholt PH Hejlesen OKM Ehlers L. Subgroup analysis of telehealthcare for patients with chronic obstructive pulmonary disease: the cluster-randomized Danish telecare north trial. ClinicoEcon Outcomes Res. (2017) 9:391–401. 10.2147/ceor.s139064 28740411 PMC5508816

[12] Jang S Kim Y Cho WK. A systematic review and meta-analysis of telemonitoring interventions on severe COPD exacerbations. Int J Environ Res Public Health. (2021) 18:6757. 10.3390/ijerph18136757 34201762 PMC8268154

[13] Lu JW Wang Y Sun Y Zhang Q Yan LM Wang YX Effectiveness of telemonitoring for reducing exacerbation occurrence in COPD patients with past exacerbation history: a systematic review and meta-analysis. Front Med (Lausanne). (2021) 8:720019. 10.3389/fmed.2021.720019 34568376 PMC8460761

[14] Wakefield BJ Scherubel M Ray A Holman JE. Nursing interventions in a telemonitoring program. Telemed J E Health. (2013) 19(3):160–5. 10.1089/tmj.2012.0098 23356382 PMC3598432

[15] Ghoulami-Shilsari F Esmaeilpour Bandboni M. Tele-nursing in chronic disease care: a systematic review. Jundishapur J Chronic Dis Care. (2019) 8(2):e84379. 10.5812/jjcdc.84379

[16] Arnaert A Delesie L. Effectiveness of video-telephone nursing care for the homebound elderly. Can J Nurs Res. (2007) 39(1):20–36. 17450703

[17] Arnaert A Delesie L. Telenursing for the elderly—the case of care via videotelephony. J Telemed Telecare. (2001) 7(6):311–6. 10.1258/1357633011936912 11747631

[18] Arnaert A Delesie L. Information visualization: a holistic tool to discover knowledge. Case study: what video-telephone care? What elderly? Knowl Manag Res Pract. (2005) 3(1):3–9. 10.1057/palgrave.kmrp.8500045

[19] Han MK Agusti A Calverley PM Celli BR Criner G Curtis JL Chronic obstructive pulmonary disease phenotypes: the future of COPD. Am J Respir Crit Care Med. (2010) 182(5):598–604. 10.1164/rccm.200912-1843CC 20522794 PMC6850732

[20] Carlsson AM. Assessment of chronic pain: I. Aspects of the reliability and validity of the visual analogue scale. Pain (1983) 16(1):87–101. 10.1016/0304-3959(83)90088-X 6602967

[21] EQ-5D-5l User Guide. Basic information on how to use the EQ-5D-5l instrument. Available at: https://www.unmc.edu/centric/_documents/EQ-5D-5l.pdf (Accessed November 10, 2018).

[22] Beekman E Verhagen A. Clinimetrics: hospital anxiety and depression scale. J Psychother. (2018) 64(2018):198. 10.1016/j.jphys.2018.04.003 29895416

[23] Emons WHM Sijtsma K Pedersen SS. Dimensionality of the hospital anxiety and depression scale (HADS) in cardiac patients. Comparison of Mokken scale analysis and factor analysis. Assessment. (2012) 19(3):337–3353. 10.1177/1073191110384951 20947706

[24] De Jong Gierveld J Van Tilburg T. DeJong-Gierveld loneliness scale. Res Aging. (2006) 28:582–98. 10.1177/0164027506289723

[25] De Jong Gierveld J Van Tilburg T. The De Jong Gierveld short scales for emotional and social loneliness: tested on data from 7 countries in the UN generations and gender issues. Eur J Ageing. (2010) 7(2):121–30. 10.1007/s10433-010-0144-6 20730083 PMC2921057

[26] Weinert C. Evaluation of the personal resource questionnaire: a social support measure. Birth Defects. (1984) 20(5):59–97. 6536339

[27] Tawalbeh L Ahmad MM. Personal resource questionnaire: a systematic review. J Nurs Res. (2013) 21(3):170–7. 10.1097/01.jnr.0000432049.31931.ab 23958606

[28] Jones PW Quirk FH Baveystock CM Littlejohns P. A self-complete measure for chronic airflow limitation—the St George’s respiratory questionnaire. Am Rev Respir Dis. (1992) 145:1321–7. 10.1164/ajrccm/145.6.1321 1595997

[29] Bourbeau J Maltais F Rouleau M Guimont C. French-Canadian version of the chronic respiratory and St George’s respiratory questionnaires: an assessment of their psychometric properties in patients with chronic obstructive pulmonary disease. Can Respir J. (2004) 11:702421. 10.1155/2004/702421 15505701

[30] Bradlow ET. Exploring repeated measures data sets for key features using principal components analysis. Available at: https://citeseerx.ist.psu.edu/viewdoc/download?doi=10.1.1.37.2187&rep=rep1&type=pdf (Accessed April 17, 2022).

[31] Gifi A. Nonlinear multivariate analysis. New York: Wiley (1990).

[32] Jolliffe IT Cadima J. Principal component analysis: a review and recent developments. Philos Trans R Soc A. (2016) 374:20150202. 10.1098/rsta.2015.0202 PMC4792409 26953178

[33] McIver JP Carmines EG. Unidimensional scaling. Beverly Hills/London: Sage University Paper Series on Quantitative Applications in Social Sciences 07-001 (1981).

[34] Jakobsen JC Gluud C Wetterslev J Winkel P. When and how should multiple imputation be used for handling missing data in randomised clinical trials—a practical guide with flowcharts. BMC Med Res Methodol. (2017) 17(1):162. 10.1186/s12874-017-0442-1 29207961 PMC5717805

[35] Murray JS. Multiple imputation: a review of practical and theoretical findings. Stat Sci. (2018) 33(2):142–59. 10.1214/18-STS644

[36] Sterne JAC White IR Carlin JB Spratt M Royston P Kenward MG Multiple imputation for missing data in epidemiological and clinical research: potential and pitfalls. Br Med J. (2009) 338:b2393. 10.1136/bmj.b2393 19564179 PMC2714692

[37] Le S Josse J Husson F. Factominer: an R package for multivariate analysis. J Stat Softw. (2008) 25(1):1–18. 10.18637/jss.v025.i01

[38] Josse J Husson F. missMDA: a package for handling missing values in multivariate data analysis. J Stat Softw. (2016) 70(1):1–31. 10.18637/jss.v070.i01

[39] Rodrigues A Camillo CA Furlanetto KC Paes T Morita AA Spositon T Cluster analysis identifying patients with COPD at high risk of 2-year all-cause mortality. Chron Respir Dis. (2019) 16:1479972318809452. 10.1177/1479972318809452 30428721 PMC6301836

[40] Kim S Lim MN Hong Y Han SS Lee SJ Kim WJ. A cluster analysis of chronic obstructive pulmonary disease in dusty areas cohort identified three subgroups. BMC Pulm Med. (2017) 17(1):209. 10.1186/s12890-017-0553-9 29246211 PMC5732468

[41] Gagnon P Casaburi R Saey D Porszasz J Provencher S Milot J Cluster analysis in patients with GOLD 1 chronic obstructive pulmonary disease. PLoS ONE. (2015) 10(4):e0123626. 10.1371/journal.pone.0123626 25906326 PMC4407903

[42] Cohen J. Statistical power analysis for the behavioral sciences. USA: Lawrence Erlbaum Associates. (1988).

[43] Cohen J. A power primer. Psychol Bull. (1992) 112(1):155–9. 10.1037//0033-2909.112.1.155 19565683

[44] Fritz CO Morris PE Richler JJ. Effect size estimates: current use, calculations, and interpretation. J Exp Psychol Gen. (2012) 141(1):2–18. 10.1037/a0024338 21823805

[45] IBM SPSS statistics 28.0.0. Available at: https://www.ibm.com/docs/en/spss-statistics/28.0.0 (Accessed April 10, 2022).

[46] AI-Therapy Statistics ^BETA^. Statistics for psychologists. Available at: https://www.ai-therapy.com/psychology-statistics/ (Accessed April 10, 2022).

[47] Psychometrica. Computation of effect sizes. Available at: https://www.psychometrica.de/effect_size.html (Accessed April 10, 2022).

[48] American Thoracic Society (ATS). St. George’s respiratory questionnaire (SGRQ). Available at: https://www.thoracic.org/members/assemblies/assemblies/srn/questionaires/sgrq.php (Accessed April 10, 2022).

[49] Monjazebi F Dalvandi A Ebadi A Khankeh HR Rahgozar M Richter J. Functional status assessment of COPD based on ability to perform daily living activities: a systematic review of paper and pencil instruments. Glob J Health Sci. (2016) 8(3):210–23. 10.5539/gjhs.v8n3p21 PMC4803967 26493419

[50] Takechi H Kokuryu A Kubota T Yamada H. Relative preservation of advanced activities in daily living among patients with mild-to-moderate dementia in the community and overview of support provided by family caregivers. Int J Alzheimer’s Dis. (2012) 2012:4148289. 10.1155/2012/418289 PMC3395251 22811947

[51] Kaiser HF. The application of electronic computers to factor analysis. Educ Psychol Meas. (1960) 20(1):141–51. 10.1177/001316446002000116

[52] Thurstone LL. Multiple factor analysis. Chicago: University of Chicago Press (1947).

[53] Kaiser HF. An index of factorial simplicity. Psychometrika. (1974) 39:31–6. 10.1007/BF02291575

[54] RStudio. RStudio: integrated development environment. Available at: http://www.rstudio.com/ (Accessed April 10, 2022).

[55] Maechler M Rousseeuw P Struyf A Hubert M Hornik K. Cluster: cluster analysis basics and extensions. R package version 2.1.3. Available at: https://CRAN.R-project.org/package=cluster (Accessed April 10, 2022).

[56] Galili T. Dendextend: an R package for visualizing, adjusting, and comparing trees of hierarchical clustering. Bioinformatics. (2015) 31(22):3718–20. 10.1093/bioinformatics/btv428 26209431 PMC4817050

[57] Kassambara A Mundt F. Factoextra: Extract and visualize the results of multivariate data analyses. R package version 1.0.7. Available at: https://CRAN.R-project.org/package=factoextra (Accessed April 10, 2022).

[58] Sullivan GM Feinn R. Using effect size-or why the p value is not enough. J Grad Med Educ. (2012) 4(3):279–82. 10.4300/JGME-D-12-00156.1 23997866 PMC3444174

[59] Serdar C C Cihan M Yücel D Serdar MA. Sample size, power and effect size revisited: simplified and practical approaches in pre-clinical, clinical and laboratory studies. Biochem Med (Zagreb). (2021) 31(1):010502. 10.11613/BM.2021.010502 33380887 PMC7745163

[60] Nordahl-Hansen A Øien RA Volkmar F Shic F Cicchetti DV. Enhancing the understanding of clinically meaningful results: a clinical research perspective. Psychiatry Res. (2018) 270:801–6. 10.1016/j.psychres.2018.10.069 30551328

[61] Ranganathan P Pramesh CS Buyse M. Common pitfalls in statistical analysis: clinical versus statistical significance. Perspect Clin Res. (2015) 6(3):169–70. 10.4103/2229-3485.159943 26229754 PMC4504060

[62] Sharma H. Statistical significance or clinical significance? A researcher’s dilemma for appropriate interpretation of research results. Saudi J Anaesth. (2021) 15(4):431–4. 10.4103/sja.sja_158_21 34658732 PMC8477766

[63] Dahlberg S Korn EL Le-Rademacher J Mandrekar SJ. Clinical versus statistical significance in studies of thoracic malignancies. J Thorac Oncol. (2020) 15(9):1406–8. 10.1016/j.jtho.2020.06.007 32580055

[64] Danne T Limbert C Domingo MP De Prato S Renard E Choudhary P Telemonitoring, telemedicine and time in range during the pandemic: paradigm change for diabetes risk management in the post-COVID future. Diabetes Ther. (2021) 12:2289–310. 10.1007/s13300-021-01114-x 34338994 PMC8327601

[65] Deloitte. COVID-19. Virtual care is here to stay (2020). Available at: https://www2.deloitte.com/content/dam/Deloitte/ca/Documents/life-sciences-health-care/ca-covid-19-digital-health-and-virtual-care-aoda-en.pdf (Accessed April 10, 2022).

[66] Bestsennyy O Gilbert G Harris A Rost J. Telehealth: a quarter-trillion-dollar post-COVID-19 reality? Available at: https://www.mckinsey.com/industries/healthcare-systems-and-services/our-insights/telehealth-a-quarter-trillion-dollar-post-covid-19-reality (Accessed April 10, 2022).

[67] Mantena S Keshavjee S. Strengthening healthcare delivery with remote patient monitoring in the time of COVID-19. BMJ Health Care Inform. (2020) 28:e100302. 10.1136/bmjhci-2020-100302 PMC8300556 34289962

[68] Christensen JKB. The emergency and unfolding of telemonitoring practices in different healthcare organization. Int J Environ Res Public Health. (2018) 15:61. 10.3390/ijerph15010061 29301384 PMC5800160

[69] Choudhary P Wilmot EG Owen K Patel DC Mills L Rayman G A roadmap to recovery: ABCD recommendations on risk stratification of adult patients with diabetes in the post-COVID-19 era. Diabetic Med. (2021) 38:e14462. 10.1111/dme.14462 33230813 PMC7744853

[70] Orso F Herbst A Migliorini M Ghiara C Virciglio S Camartini V Telehealth management and risk stratification of older patients with chronic heart failure during COVID-19 pandemic: prognostic evaluation of the TeleHFCovid19-score. JAMDA. (2022) 23:421–7. 10.1016/j.jamda.2021.12.024 35041828 PMC8702408

[71] Nikolaou V Massor S Fakhimi M Stergioulas L Price D. COPD Phenotypes and machine learning cluster analysis: a systematic review and future research agenda. Respir Med. (2020) 171:106093. 10.1016/j.rmed.2020.106093 32745966

[72] Chai CS Mos S Ng DLC Goh GMKC Su AT Ibrahim MAB Clinical phenotypes and heath-related quality of life of COPD patients in a rural setting in Malaysia—a cross-sectional study. BMC Pulm Med. (2020) 20(1):254. 10.1186/s12890-020-01295-4 32993591 PMC7526228

[73] Pariksh R Jain SH Navathe AS. The sociobehavioral phenotype: applying a precision medicine framework to social determinants of health. Am J Manag Care. (2019) 25(9):421–3. 31518090

[74] IBM SPSS Statistics Guides. Cluster analysis. Availbale at: https://www.norusis.com/pdf/SPC_v19.pdf (Accessed April 10, 2022).

[75] Hackshaw A. Small studies: strengths and limitations. Eur Respir J. (2008) 32(5):1141–3. 10.1183/09031936.00136408 18978131

[76] Brydges CR. Effect size guidelines, sample size calculations, and statistical power in gerontology. InnovAging. (2019) 3(4):igz036. 10.1093/geroni/igz036 PMC6736231 31528719

[77] Ibrahim JG Chu H Chen MH. Missing data in clinical studies: issues and methods. J Clin Oncol. (2012) 30(26):3297–303. 10.1200/JCO.2011.38.7589 22649133 PMC3948388

